# Antibiotic prescription practices and attitudes towards the use of antimicrobials among veterinarians in the City of Tshwane, South Africa

**DOI:** 10.7717/peerj.10144

**Published:** 2021-01-13

**Authors:** Ronita Samuels, Daniel Nenene Qekwana, James W. Oguttu, Agricola Odoi

**Affiliations:** 1 Biomedical and Diagnostic Sciences, College of Veterinary Medicine, The University of Tennessee, Knoxville, TN, USA; 2 Section of Veterinary Public Health, Department of Paraclinical Sciences, Faculty of Veterinary Science, University of Pretoria, Pretoria, South Africa; 3 Department of Agriculture and Animal Health, College of Agriculture & Environmental Sciences, University of South Africa, Johannesburg, South Africa

**Keywords:** Antimicrobial resistance, South Africa, City of Tshwane, Prescription practices, Judicious antimicrobial use, Antimicrobial stewardship

## Abstract

**Background:**

Understanding the prescription practices and attitudes of veterinarians towards antimicrobial resistance (AMR) is crucial in guiding efforts to curb AMR. This study investigated prescription practices and attitudes towards AMR among veterinarians in the City of Tshwane, South Africa.

**Methods:**

Out of the 83 veterinarians invited to participate in the study, 54 signed the consent form and completed the questionnaire. Percentages and 95% confidence intervals of all categorical variables were computed. A multinomial logistic model was used to identify predictors of the veterinarians’ view towards antimicrobial use.

**Results:**

The majority (88%) of respondents indicated that improper use of antimicrobials contributed to selection for AMR. Veterinarians relied on clinical signs and symptoms (88%, 48/54) to decide whether to prescribe antimicrobials or not. However, the choice of antimicrobials depended on the cost of antibiotics (77.2%), route of administration (81.5%), and risk of potential adverse reactions (79.6%; 43/54). Many (61.5%) veterinarians were of the view that often antimicrobials are appropriately prescribed and 88.7% agreed that improper use of antimicrobials contributed to selection for antimicrobial resistant organisms. Compared to females, males were significantly more likely (Relative Risk Ratio (RRR) = 9.0; *P* = 0.0069) to agree rather than to “neither agree nor disagree” that their colleagues over-prescribed antimicrobials.

**Conclusions:**

The decisions to prescribe antimicrobials by the veterinarians depended on clinical presentation of the patient, while the choice of antimicrobial depended on cost, route of administration, and risk of potential adverse reactions. Most veterinarians were of the view that antimicrobials were prescribed judiciously.

## Introduction

Due to a combination of factors, but most notably the rise in use of antibiotics to treat both human and domestic animals, antimicrobial resistance (AMR) has become a global scientific and public health concern (Bryan et al., 2010; Maddox et al., 2015). Available evidence suggests that widespread and indiscriminate use of antimicrobials in animals fosters the emergence of zoonotic pathogens that exhibit AMR (Nikaido, 2009; Zishiri, Mkhize & Mukaratirwa, 2016). The development and spread of antimicrobial resistant pathogens impede both preventative and therapeutic uses of antibiotics. This problem is becoming increasingly important in low-income African countries (Adefisoye & Okoh, 2016). Levels of AMR vary greatly between countries, and this could be attributed to differences in antimicrobial prescription practices of both medical and veterinary practitioners (Ahmed et al., 2010). Moreover, inappropriate use of antimicrobials is said to be one of the common factors driving development of AMR (Quet, Newton & Longuet, 2015).

Although several public health studies in the United States, China, Democratic Republic of Congo, and Italy have assessed the knowledge and attitudes of medical students regarding AMR (Abbo et al., 2013; Huang et al., 2013; Thriemer et al., 2013; Scaioli et al., 2015), studies investigating attitudes of veterinarians towards antimicrobial prescription practices and usage are limited. In addition, the few available veterinary studies have largely focused on the antimicrobial prescription habits of veterinarians in developed countries (De Briyne et al., 2013; Postma et al., 2016; Carmo et al., 2018; Van Cleven et al., 2018; Hopman et al., 2018). Although one South African study (Chipangura et al., 2017) investigated antimicrobial usage patterns of small animal veterinarians, there is no evidence of any studies that have investigated the attitude of veterinarians towards antimicrobial use and AMR in South Africa. Moreover, one in five medications (including antibiotics) on the South African market, are counterfeit (Moyane, Jideani & Aiyegoro, 2013) and most veterinarians do not always follow antimicrobial prescription policies (Chipangura et al., 2017).

Therefore, the objectives of this study were to: (a) assess the knowledge, antimicrobial prescription practices, and attitudes towards AMR among veterinarians in the City of Tshwane Metropolitan Municipality; (b) investigate factors associated with the view of veterinarians regarding antimicrobial use and its potential impact on development of AMR. The information generated from this study will help guide programs to slow down and/or curb the development of AMR.

## Materials and Methods

### IRB/Ethics approval

The study was approved by the Ethics Review Boards of both the University of Tennessee (number: 619622) and the University of South Africa (number: 2017/CAES/017). The management of the veterinary clinics that agreed to participate in the study granted permission to distribute the survey among the veterinary clinicians. Written consent was obtained from study respondents before they completed the questionnaire. Data collection was performed between April and July 2017.

### Study area

This cross-sectional questionnaire survey collected data from practicing veterinarians in the City of Tshwane Metropolitan Municipality, South Africa. The City of Tshwane Metropolitan Municipality is among the five administrative areas in Gauteng Province of South Africa. The municipality is 6,368 square kilometers with a population of 2,921,500.

Under the Veterinary and Para-veterinary Professions Act 19 of 1982, all practicing veterinarians in South Africa are required to register with the South African Veterinary Council (SAVC). All 56 registered veterinary clinics in the City of Tshwane Municipality were approached, but only 28 agreed to participate in the study. The clinics were identified using the database of the South African Veterinary Association and SAVC, and Google Maps used to identify their geographic locations.

### Data collection

A 30-item questionnaire, adapted from two previous survey questionnaires developed by Hughes et al. (2012) and Jacob et al. (2015), was used to collect data on the attitudes and antimicrobial prescription practices of veterinarians in the study area. The original questionnaires (Hughes et al., 2012; Jacob et al., 2015), which had questions on the opinion of clinical veterinarians regarding antimicrobial use and antimicrobial-resistant infections as well as antimicrobial prescribing patterns, were modified by adding questions on prescription practices, attitudes towards prescription practices, and AMR. The questionnaire was pretested on a small sample of clinical veterinarians at the Faculty of Veterinary Medicine, University of Pretoria. This allowed identification and correction of ambiguous or misleading questions and addition of response categories previously omitted.

The final questionnaire was designed to take 20–30 min to complete and covered areas related to the attitude of veterinarians towards antimicrobial prescription practices and how their prescription practices related to the development of AMR. The questions were grouped into six sections: demographics, veterinary education, antimicrobial prescription practices, factors associated with prescribing habits, opinions about prescription practices, and opinions about AMR. The six sections contained both open-ended and close-ended questions consisting of a combination of yes/no questions, multiple choice questions as well as a 5-point Likert scale (ranging from “strongly agree” to “strongly disagree”).

An online version of the questionnaire was uploaded onto Qualtrics (Qualtrics, Provo, UT, USA, 2013), and participants were provided with a web link to access the survey and provide responses anonymously. Additionally, the survey questionnaire was printed and distributed in person to 28 clinics that agreed to participate in the study. To improve the response rate reminder e-mails were sent to potential survey respondents. Phone calls were also made to encourage them to complete the survey. In addition, all the clinics that did not respond to the survey were also visited. For facilities that had more than one veterinarian, all of them were requested to complete the questionnaire. A total of 83 survey questionnaires were distributed and 54 were completed and returned.

### Data management and analysis

#### Data management

Data from the completed questionnaire were entered into Microsoft Excel (Microsoft Corporation, Redmond, WA, USA). The dataset was assessed for inconsistencies and missing values. Due to the small number of responses to some categories of some of the questions, some responses that required 5-point Likert scale responses were re-categorized as follows: “strongly agree” and “agree” were re-categorized into “agree” while “strongly disagree” and “disagree” into “disagree”.

#### Data analysis

All statistical analyses were performed in SAS 9.4 (2012; SAS Institute,Cary, NC, USA). The distributions of demographic variables and their 95% confidence intervals were computed and presented as tables. Shapiro–Wilk test of normality was used to evaluate the distributions of continuous variables (number of years of work experience, years since graduation, and the number of veterinarians working or employed at any given practice). These variables were found to be non-normally distributed and hence medians and interquartile ranges were reported.

A multinomial logistic regression model was used to investigate predictors of “Your colleagues over-prescribe antimicrobials” as the outcome variable. This outcome variable had three possible responses: “agree”, “neither agree nor disagree” and “disagree”. The response category “neither agree nor disagree” was used as the baseline (referent) level and hence Relative Risk Ratio (RRR) estimates were computed for “agree” and “disagree” both of which were compared to the “neither agree nor disagree”. The model building process was done in two steps: The first step entailed building a univariable logistic regression model to investigate the relationships between each potential predictor and the outcome. Potential predictors with *P*-values ≤ 0.20 were considered for inclusion in a multivariable regression model. In the 2nd step, a multivariable model was fit using manual backward selection. Statistical significance was assessed at α ≤ 0.05.

Confounding was assessed by comparing the change in model coefficients with and without the suspected confounders. If the removal of a suspected confounding variable resulted in a 20% or greater change in the coefficient of another variable, the variable that was removed was considered a confounder and retained in the model regardless of its statistical significance (Dohoo, Martin & Stryhn, 2009, 2012). However, no confounders were identified.

Relative risk ratios as well as their 95% confidence intervals were computed for predictor variables retained in the final multinomial model. Goodness-of-fit of the final multinomial model was assessed by fitting ordinary logistic regression models to each pairwise combination of the three potential outcome categories as recommended by Dohoo, Martin & Stryhn (2003). Hosmer–Lemeshow goodness-of-fit tests were then used to assess the fit of each of the binomial models separately. The reason for adopting this approach was that currently there are no available multinomial model fit assessment tests in SAS, the statistical software used in this study.

## Results

### Respondent information

A total of 83 potential respondents were invited to participate in the study, 54 of whom completed the survey resulting in a response rate of 65% (54/83). Of the 54 completed questionnaire surveys, eight were completed online while 46 were paper copies.

Of the 54 veterinarians who participated in the study, 53.7% (95% CI [39.6–67.4]; 29/54) were females and 46.3% (95% CI [32.6–60.4]; 25/54) were males. Most of the respondents (71.7%, 95% CI [57.7–83.2]; 38/53) were in small animal practice, while the rest (28.3%, 95% CI [16.8–42.4]; 15/53) were in mixed animal practice. More than half (55.6%, 95% CI [41.4–69.1]; 30/54) of the veterinarians worked at veterinary hospitals while the remaining 44.4% (95% CI [30.9–58.6]; 24/54) worked at veterinary clinics. Forty-three percent (42.6%, 95% CI [29.2–56.8]; 23/54) of the respondents indicated that they had completed postgraduate training. The median number of years of work experience of the respondents was three years (Interquartile Range (IQR): 2, 7) while the median years since graduation was 10 years (IQR: 0, 26). The median number of veterinarians working or employed at any given practice was four (IQR: 1, 14).

### Training on antimicrobials during veterinary education

Over half (55.6%; 30/54) of the veterinarians that were interviewed indicated that antibiotics and their use were emphasized in multiple courses during the pre-clinical years of their veterinary education, while 64.8% (35/54) indicated that antibiotics were emphasized only in courses taught during the clinical years of their veterinary training. According to most respondents (72.2%; 39/54), pharmacologists and clinical pharmacologists were responsible for training related to antibiotics during their clinical years (Table 1).

**Table 1:** Antibiotics training among veterinarians in the City of Tshwane Municipality, South Africa (2017).

Question/Response	Number	Percent	95% CI^1^
**What was the emphasis on antibiotics in veterinary school education (non-clinical years)?**	**54**		
Topic was not covered	0	0.0	[0.0–6.6]
Light emphasis	4	7.4	[2.1–17.9]
Covered thoroughly in one course	20	37.0	[24.3–51.3]
Emphasized in multiple courses	30	55.6	[41.4–69.1]
**What was the emphasis on antibiotics in your veterinary school education (clinical years)?**	**54**		
Topic was not covered	0	0.0	[0.0–6.6]
Light emphasis	12	22.2	[12.0–35.6]
Covered thoroughly in one course	7	13.0	[5.4–24.9]
Emphasized in multiple courses	35	64.8	[50.6–77.3]
**What was the background of the person primarily responsible for your education on antibiotics during your veterinary education?^2^**	**54**		
Clinical pharmacist	7	13.0	[5.4–24.9]
Clinical microbiologist	6	11.1	[4.2–22.6]
Clinician	16	29.6	[18.0–43.6]
Pharmacologist/clinical pharmacologist	39	72.2	[58.4–83.5]
Toxicologist	4	7.4	[2.1–17.9]
Do not know his/her background	1	1.9	[0.1–9.9]
**What are the main sources that you use to receive current information on antimicrobials and their use?^2^**	**54**		
Practice policy	13	24.0	[13.5–37.6]
Pharmaceutical companies	24	44.4	[30.9–58.6]
Veterinary medicine directorates	12	22.2	[12.0–35.6]
Peer reviewed scientific literature	30	55.6	[41.4–69.1]
Textbook/drug handbook	44	81.5	[68.6–90.8]
Continuing professional development courses	38	70.4	[56.4–82.0]

**Notes:**

1 95% Confidence Interval.

2 Percentages sum up to >100% because some respondents selected more than one response.

The majority of respondents indicated that the most common sources of information on antimicrobials and antimicrobial use were textbooks/drug handbooks (81.5%; 44/54), followed by continuing professional education courses (70.4%; 38/54), peer reviewed scientific literature (55.6%; 30/54) and pharmaceutical companies (44.4%; 24/54). Only 24% (13/54) of the veterinarians indicated that antimicrobial use policies at their work places were the main sources of information on antimicrobials and antimicrobial usage (Table 1).

### Antimicrobial prescription practices

The majority (92.3%; 48/52) of the veterinarians indicated that they were able to prescribe antimicrobials without oversight from a supervisor. As many as 79.2% (38/48) of the respondents indicated that they do prescribe antimicrobials multiple times a day, but only 39.6% (21/53) reported that they were not comfortable with prescribing certain antibiotics. When asked what factors influenced the choice of antimicrobials to prescribe when there was a need for it, a number of respondents (72.2%; 39/54) indicated that the cost of antibiotics was the main influencing factor. Other factors they cited were route of administration (81.5%; 44/54), and risk of potential adverse drug reactions (79.6%; 43/54) (Table 2).

**Table 2:** Prescription practices among veterinarians in the City of Tshwane Municipality, South Africa (2017).

Question/Responses	Number	Percentage	95% CI^1^
**Can you prescribe antibiotics without supervision, approval, or additional oversight?**	**52**		
Yes	48	92.3	[81.5–97.9]
No	4	7.7	[2.1–18.5]
**On average, how often do you prescribe antibiotics?**	**48**		
Multiple times per day	38	79.2	[65.0–89.5]
Once per day	4	8.2	[2.3–20.0]
Once every 2 days	2	4.2	[0.5–14.3]
Once per week	2	4.2	[0.5–14.3]
Once per month	2	4.2	[0.5–14.3]
**Is there any antibiotic that you do not feel comfortable prescribing?**	**53**		
Yes	21	39.6	[26.5–54.0]
No	32	60.4	[46.0–73.6]
**Do any of the factors below affect your decision when choosing to prescribe an antibiotic to a patient?^2^**	**54**		
Cost of antibiotic	39	72.2	[58.4–83.5]
Client insurance	2	3.7	[0.5–12.8]
Client expectations	9	16.7	[7.9–29.3]
Route of administration	44	81.5	[68.6–90.8]
Frequency of patient visits	16	29.6	[17.9–43.6]
Risk of potential adverse drug reaction	43	79.6	[66.5–89.37]
**You always rely on clinical signs and symptoms when prescribing an antibiotic**	**54**		
Agree	48	88.9	[77.4–95.8]
Neither agree nor disagree	2	3.7	[0.5–12.8]
Disagree	4	7.4	[2.1–17.9]
**You rely on laboratory results before prescribing an antibiotic**	**53**		
Agree	23	43.4	[29.8–57.8]
Neither agree nor disagree	15	28.3	[16.8–42.3]
Disagree	15	28.3	[16.8–42.3]
**What are your feelings concerning antibiotic prescription at your facility or practice?**	**52**		
Some antibiotics are under-prescribed	6	11.5	[4.4–23.4]
All antibiotics are appropriately prescribed	32	61.5	[47.0–74.7]
Some antibiotics are over-prescribed	14	27.0	[15.6–41.0]
**Do you feel like you sometimes over-prescribe antibiotics?**	**53**		
Yes	17	32.1	[19.9–46.3]
No	36	67.9	[53.7–80.1]
**Your colleagues over-prescribe antibiotics**	**53**		
Agree	20	37.8	[18.3–44.3]
Neither agree nor disagree	18	33.9	[21.5–48.3]
Disagree	15	28.3	[16.8–42.3]
**Does your veterinary facility or practice have a policy concerning antibiotic prescription?**	**51**		
Yes	35	68.6	[54.1–80.9]
No	16	31.4	[19.1–45.9]
**Veterinarians at your practice or facility you always comply with antibiotic prescription policies**	**53**		
Agree	28	52.8	[29.8–57.7]
Neither agree nor disagree	16	30.2	[18.3–44.3]
Disagree	9	17.0	[8.0–29.8]

**Notes:**

1 95% Confidence Interval.

2 Percentages sum up to >100% because some respondents selected more than one response.

Regarding what guided the veterinarians’ decisions to prescribe antimicrobials, most of the respondents (88.9%, 48/54) agreed that they always relied on clinical signs and symptoms to prescribe antimicrobials. On the other hand, 43.4% (23/53) of the respondents agreed that they prescribe antibiotics based on antibiogram, and only 28.3% (15/53) disagreed (Table 2).

### Attitude towards antimicrobial prescription

With respect to judicious or injudicious use of antimicrobials, many (61.5%; 32/52) of the veterinarians were of the view that often antimicrobials were appropriately prescribed. However, 27% (14/52) indicated that some antimicrobials tended to be over-prescribed. Regarding their prescription practices and those of their colleagues, only 32.1% (17/53) of the respondents admitted to sometimes over-prescribing antimicrobials while 37.8% (20/53) agreed that their colleagues tended to over-prescribe antimicrobials. Regarding prescription policies, 68.6% (35/51) of the respondents indicated that their facility had antibiotic prescription policy while 52.8% (28/53) agreed that the colleagues at their practice complied with antimicrobial prescription policies.

### Attitude towards antimicrobial resistance

Regarding how the presence of antimicrobial prescription policy influenced prescription practices, only 39.7% (21/53) of the respondents agreed that the presence of antimicrobial prescription policy influenced the incidence of AMR at their facility. In response to the question regarding improper use of antimicrobials and its role in the development of resistance, 88.7% (47/53) of the respondents agreed that improper use of antimicrobials contributed to selection for antimicrobial resistant organisms. However, regarding whether the improper use of antimicrobials by their colleagues contributed to AMR, half of the respondents (50.9%; 27/53) neither agreed nor disagreed that improper use of antimicrobials by their colleagues contributed to selection for antimicrobial resistant organisms at their facility. Regarding the status of AMR at the facility where they worked, only 24.1% (13/52) of the veterinarians agreed that there had been an increase in the incidence of AMR cases at their practice (Table 3).

**Table 3:** Opinions on antimicrobial resistance among veterinarians in the City of Tshwane, South Africa (2017).

Question/Response	Number	Percentage	95% CI^1^
**Antibiotic prescription policies are contributing to a change in the frequency of antimicrobial resistance at your facility or practice**	**53**		
Agree	21	39.7	[19.9–46.3]
Neither agree nor disagree	23	43.4	[29.8–57.7]
Disagree	9	16.9	[8.1–29.8]
**Improper use of antibiotics contributes to selection for antimicrobial resistance**	**53**		
Agree	47	88.7	[13.8–38.3]
Neither agree nor disagree	2	3.8	[0.5–13.0]
Disagree	4	7.5	[2.1–18.2]
**Improper prescribing habits among your colleagues is affecting the selection for antibiotic resistance in your facility**	**53**		
Agree	17	32.1	[20.0–46.3]
Neither agree nor disagree	27	50.9	[36.8–64.9]
Disagree	9	17.0	[8.1–29.8]
**There has been an increase in the number of cases of antimicrobial resistance at your facility or practice**	**54**		
Agree	13	24.1	[13.5–37.6]
Neither agree nor disagree	18	33.3	[21.1–47.5]
Disagree	23	42.6	[29.2–56.8]

**Note:**

1 95% Confidence Interval.

### Predictors of the tendency to over prescribe antimicrobials by veterinarians

The tendency to over prescribe was assessed using the question; “Do your colleagues over-prescribe antimicrobials”. A significant association was observed in the univariable models between the polytomous dependent variable (Your colleagues over-prescribe antimicrobials) and each of the independent variables gender (*P* = 0.007), veterinary practice (*P* = 0.178), and veterinary facility (*P* = 0.166) at a relaxed *P*-value of ≤0.2. As a result, these variables were assessed in the multivariable multinomial model (Table 4).

**Table 4:** Univariable and final multinomial logistic models investigating predictors of “Do your colleagues over-prescribe antimicrobials”.

Variable	Number	Univariable multinomial models						Final multinomial model					
		Agree			Disagree			Agree			Disagree		
		RRR^1^	95% CI^2^	*P*-value	RRR^1^	95% CI^2^	*P*-value	RRR^1^	95% CI^2^	*P*-value	RRR^1^	95% CI^2^	*P*-value
**Gender**	**51**												
Male	24	10.5	[2.3–47.2]	0.0022	2.2	[0.5–10.6]	0.3303	9.0	[1.8–44.7]	0.0069	1.7	[0.3–9.6]	0.5346
Female	27	ref.	–	–	–	–	–	ref.	–	–	–	–	–
**Veterinary practice**	**53**												
Mixed	15	0.3	[0.1–1.2]	0.0868	0.3	[0.1–1.7]	0.1843						
Small Animal	38	ref.	–	–	–	–	–						
**Veterinary facility**	**54**												
Veterinary Clinic	30	0.3	[0.1–1.3]	0.1118	0.3	[0.1–1.3]	0.1015						
Veterinary Hospital	24	ref.	–	–	–	–	–						
**Years of experience**	**53**												
≥4 years	26	1.5	[0.4–5.5]	0.5166	1.8	[0.4–7.7]	0.4577						
≤3 years	27	ref.	–	–	–	–	–						
**Hours worked per week**	**51**												
≤44 h	25	0.6	[0.2–2.2]	0.4209	1.8	[0.4–8.1]	0.4755						
≥45 h	26	ref.	–	–	–	–	–						
**Years since graduation**	**54**												
6–10 years	11	2.3	[0.3–16.2]	0.3911	2.3	[0.3–16.2]	0.3911						
≥11 years	26	2.6	[0.6–12.0]	0.2132	1.1	[0.2–5.8]	0.9158						
≤5 years	17	ref.	–	–	–	–	–						
**Antibiotic Policy**	**51**												
Yes	16	1.2	[0.3–4.8]	0.8126	1.0	[0.2–4.8]	0.9778						
No	35	ref.	.	.	.	.	.						

**Notes:**

1 Relative Risk Ratios.

2 95% Confidence Interval.

In the final model, only gender was a significant predictor of “Do your colleagues over-prescribe antimicrobials”. Male respondents compared to female respondents were significantly more likely (RRR = 9.0; *P* = 0.0069) to agree that their colleagues over-prescribe antimicrobials rather than to neither agree nor disagree (Table 4). In this model, the categories “agree” and “disagree” are each compared to “neither agree or disagree” because the latter was the referent level used in the multinomial model.

## Discussion

This study used a questionnaire survey to investigate prescription practices and attitudes towards AMR among veterinarians in the City of Tshwane Metropolitan Municipality, South Africa. Although past studies investigated knowledge and perceptions of medical and pharmacy students towards antimicrobials and AMR (Ahmad et al., 2015; Scaioli et al., 2015; Haque et al., 2016; Anyanwu et al., 2018; Dyar et al., 2018; Seid & Hussen, 2018), few studies have focused on opinions and attitudes on antimicrobial use and stewardship in the veterinary profession (Hardefeldt et al., 2018a, 2018b) and examined the breadth of training on antimicrobials during both pre-clinical and clinical years of veterinary education (Dyar et al., 2018; Hardefeldt et al., 2018b). Therefore, this study is intended to fill this knowledge gap.

### Training on antibiotics during veterinary education

A large percentage of the respondents in this study indicated that antibiotics were emphasized or covered thoroughly in one or multiple courses at pre-clinical and clinical levels. In addition, pharmacologists and clinical pharmacologists were responsible for training related to antibiotics during their clinical years. Furthermore, to improve their knowledge of antimicrobials and antimicrobial use, veterinarians mostly used textbooks/drug handbooks, attended continuing professional education courses, read peer reviewed scientific literature, and consulted pharmaceutical companies. These findings are similar to those of an Australian study which reported that veterinarians attended conferences or meetings, used self-directed education and webinars or podcasts to improve their knowledge of antimicrobial prescriptions and use (Hardefeldt et al., 2018a).

A relatively small percentage (24%) of veterinarians in this study indicated that they consulted antimicrobial prescription policies at their practices. Although these findings are similar to those reported by Chipangura et al. who indicated that most veterinarians did not always follow antimicrobial prescription policies (Chipangura et al., 2017), it was contrary to our expectation given that most respondents (68.6%) in the current study indicated that their practice had antimicrobial prescription policy. This might suggest that antimicrobial prescription policies are developed but not actively used to enforce judicious use of antimicrobials in clinics and hospitals. Furthermore, it is concerning that up to 31.4% of veterinary practices in this study did not have an antimicrobial prescription policy. This needs to be addressed since it is well known that failure to implement prescription policies is associated with injudicious use of antimicrobials (Mateus, Brodbelt & Stärk, 2011; De Briyne et al., 2014; Mateus et al., 2014). Therefore, there is need to encourage more practices to establish and enforce the application of antimicrobial prescription policies so as to curb the development of AMR. Contrary to the findings of this study that the majority of the practices (68.6%) had antimicrobial prescription policies, an Australian study reported that veterinary practices rarely (15%) had antimicrobial prescription policies (Hardefeldt et al., 2018a). It is apparent that there is need for improvement in terms of development of AMR policies.

### Antimicrobial prescription practices

Most (79.2%) veterinarians indicated that they prescribed antimicrobials multiple times a day and their decisions to prescribe antimicrobial were largely influenced by the clinical presentation of the patient. However, the choice of antimicrobial to use depended mainly on the cost, route of administration, and risk of potential adverse reaction. These findings are similar to those of a study by Mateus et al. (2014) who reported that cost, clinical signs, and route of administration were important factors considered by veterinarians when deciding to prescribe antimicrobials. An Australian study by Hardefeldt et al. (2018a) also reported that cost was as an important factor in the decision regarding antimicrobial prescription indicating that cost of the antimicrobial is an important consideration regardless of the geographical location. However, the cost and affordability of medications is especially important in low-income settings where prescribers often choose cheaper alternatives (Kpokiri, Taylor & Smith, 2020).

Less than half (43%) of the veterinarians in this study indicated that they relied on laboratory results when deciding the antimicrobial to prescribe. This was much lower than that of another South African study which reported that 91% of the veterinarians used antimicrobials empirically before requesting for laboratory testing (Chipangura et al., 2017). A similar survey in Greece reported that 73% of the respondents initiated empirical treatment while waiting for laboratory results or that they used antibiogram only when the treatment was unsuccessful (Valiakos et al., 2020). It is important to note that the practice of not waiting for the results of the antibiogram before implementing an antimicrobial treatment regime is not uncommon in veterinary medicine (Wayne, McCarthy & Lindenmayer, 2011). For example, Fowler et al. (2016) observed that in the US, only 36% of veterinarians surveyed chose to order culture and sensitivity tests before treating suspected bacterial infections. In Italy, the situation is even worse, with Barbarossa et al. (2017) reporting that only 7% of veterinarians who participated in their study, ordered for culture and sensitivity tests before implementing antimicrobial treatment. It is therefore evident that there is a need to educate veterinarians on the importance of use of antibiogram before making antibacterial treatment decisions. However, it is worth pointing out that in low- and middle-income countries (LMIC) there is usually lack of laboratories to perform the necessary tests and access is an important limiting factor (Petti et al., 2006; Kpokiri, Taylor & Smith, 2020). Therefore, improvement of access to laboratory facilities in such situations would play an important role in the fight against AMR. Since reliable diagnostic testing is severely limited in sub-Saharan Africa, making laboratory testing more available to guide clinical decisions and judicious use of antibiotics needs to be a priority (Petti et al., 2006; Kpokiri, Taylor & Smith, 2020). Suffice it to say that addressing the problem of AMR will require: (a) ensuring availability of diagnostic testing, (b) providing education to healthcare and veterinary professions as well as the general public, (c) development/improvement of regulations and audit on production, distribution and dispensing of drugs, (d) improving interaction between policy makers, academia, medical/veterinary professionals and civil society and (e) designing and studying easy and scalable interventions (Cox et al., 2017).

### Attitudes towards antimicrobial prescription practices

Most (88.7%) respondents in this study agreed that improper use of antimicrobials contributed to selection for antimicrobial resistant organisms. This is higher than the 50% reported by Hardefeldt et al. (2018a) in Australia. It is interesting to note that veterinarians in mixed animal practices, compared to those in small animal practices, were less likely to agree that improper use of antimicrobials contributed to selection for AMR. The reason for this is unclear and warrants further investigation. However, only 32.1% of the respondents agreed that improper use of antimicrobials by their colleagues contributed to AMR. This suggests that the majority of veterinarians who were interviewed in this study were of the view that their colleagues were not responsible for injudicious use of antimicrobials or that if they were involved in injudicious use of antimicrobials, the practice did not lead to AMR. Males, compared to females, were more likely to believe that antimicrobial use (AMU) contributes to development of AMR. The reason for this is unclear but may be because males are more likely to prescribe antimicrobials than females (Eggermont et al., 2018) and increased AMU increases risk of development of AMR. Although the study by Eggermont et al. (2018) investigated AMU among physicians, it is possible that this gender difference might apply to veterinarians as well and hence may explain the perceived differences in the belief that AMU contributes to development of AMR. However, further investigations are warranted to elucidate these differences.

Twenty seven percent of the veterinarians in this study were of the view that some antimicrobials were over-prescribed which is less than the 51.6% reported among the veterinarians in Tennessee, United States (Ekakoro & Okafor, 2019) and 88% reported among veterinarians in North Carolina, United States (Jacob et al., 2015). This may be related to higher availability of antimicrobials and ability of animal owners to pay for antimicrobials in a more developed economy like the United States compared to a middle-income economy such as South Africa. Information regarding adherence of South African veterinarians to judicious use of antimicrobials is scarce. However, a South African medical record review reported that overall guideline adherence was only 45.1% and that the main reasons for non-adherence were an undocumented diagnosis (30.5%), antibiotic not required (21.6%), incorrect dose (12.9%), incorrect drug (11.5%), and incorrect duration of therapy (9.5%) (Gasson, Blockman & Willems, 2018). Another South African report indicated that 78% of patients cared for in public clinics and 67% of those in private general practices received antibiotics, even though antibiotics were not clinically indicated. Doctors and nurses indicated that the reasons for their unnecessary antibiotic prescription were that patients demanded or expected antibiotics. However, over 50% of the patients still received antibiotics even after stating they didn’t want them unless they were necessary for treatment of their condition (Wits University, 2019). Studies are warranted to assess these in veterinary practices in South Africa and other countries.

### AMR and antimicrobial stewardship policies in South Africa

Poverty is a major driver of the development of AMR in both developing and developed countries (Planta, 2007). In developing countries, factors such as inadequate access to effective drugs, unregulated dispensing and manufacture of antimicrobials, and incomplete antimicrobial treatments due to cost are contributing to the development of AMR (Planta, 2007). The Global Action Plan on AMR calls for the use of antimicrobial medicines in human and animal health to be optimized, in tandem with a strengthening of the knowledge and evidence base through surveillance and research (Schellack et al., 2017). The South African Society of Clinical Pharmacy provides guidelines for various approaches to antibiotic preservation, behavioral change, stewardship measures, and monitoring strategies (Schellack et al., 2018). However, improved policing of these guidelines will be necessary to slow the development of AMR. A scoping review of published literature on antimicrobial stewardship (AMS) in South Africa suggested that AMS interventions should be addressed using a number of strategies: (i) prescription audits and usage; (ii) education and its impact; and (iii) the role of different healthcare professionals in AMS (Chetty et al., 2019). The report concluded that there is value for AMS in both the public and private health sectors of South Africa and that initiatives are being carried out across both sectors but more attention needs to be focused on AMS implementation in line with the National AMR Strategy of South Africa. The authors report that collaboration between the different sectors will aid in overcoming the AMR challenge (Chetty et al., 2019). An Australian study of companion animal, equine, and bovine veterinarians reported that veterinary practices rarely had antimicrobial prescribing policies (Hardefeldt et al., 2018a). They reported that the key barriers to implementation of AMS programs were: (1) a lack of AMS governance structures, (2) client expectations and competition between practices, (3) cost of microbiological testing, and (4) lack of access to education, training and AMS resources (Hardefeldt et al., 2018a). These challenges are similar to those in LMICs such as South Africa.

Some studies have recommended keeping good dispensing records that can be audited by professional peers (Tangcharoensathien, Chanvatik & Sommanustweechai, 2018) to help improve judicious use of antimicrobials. Banning the use of antibiotics as growth promoters, and continuing professional development training have also been recommended to curb the problem and may be worth considering in the South African situation (Tangcharoensathien, Chanvatik & Sommanustweechai, 2018). On the medical front, South Africa has established the South African Antimicrobial Resistance National Strategy framework to provide a structure for managing AMR, limit further increases in resistant microbial infections, and improve patient outcomes (Department of Health, Republic of South Africa, 2014). However, a One Health strategy may need to be considered to address the problem in both human and veterinary medical practices.

The South African Antibiotic Stewardship Program, a multidisciplinary expert group, has been working to implement antibiotic stewardship programs across primary and secondary care. Their activities have been supported by the South Africa’s National Department of Health through the publication of a national strategy document which defines a number of objectives, including the promotion of appropriate antibiotic use. National guidelines for antibiotic prescription exist in South Africa and are available electronically, but they do not apply to the private sector, where prescriptions are based largely on the clinical evaluation of the practitioner (Chunnilall et al., 2015), although there may be some carry over because most practitioners working in the private sector also work in the public sector (Krockow & Tarrant, 2019).

Evidence on effective and feasible stewardship interventions in LMICs is limited, and challenges for implementation of interventions are numerous (Cox et al., 2017). Therefore, strategic points might need to be progressively addressed in LMICs, such as (a) ensuring availability of diagnostic testing, (b) providing dedicated education on AMR both for healthcare workers and the general public, (c) improving regulations and audit on production, distribution and dispensing of drugs, and (d) synergism between policy makers, academia, professional bodies and civil society (Cox et al., 2017).

Policy-makers need to encourage health systems to change from providing easy access to antimicrobials to encouraging appropriate use of antimicrobials so as to reduce the risk of resistance. This is a particular challenge for LMICs that have pluralistic health systems where antibiotics are available in a number of different markets (Merrett et al., 2016). One of the strategies to address the problem is behavior change focusing on antibiotic prescribing, dispensing, use, and handling. There is evidence that unnecessary use of antibiotics is influenced by several factors including: (a) Individual factors: knowledge, attitudes, and beliefs; (b) Interpersonal factors: social identity, support, roles; (c) Institutional: rules, guidelines, regulations, and informal structures; (d) Community: social networks, norms; and (e) Public policy: regulations and laws (Lundborg & Tamhankar, 2014). Therefore, focusing on these key areas is essential. It is also important that there are synergies between interventions addressing access strategies, antibiotic quality, and diagnostics for low-resource settings. Suffice it to say that successful integration of the different strategies will require effective governance and partnerships at the national, regional and global levels (Merrett et al., 2016).

### Counterfeit medications

Not much information is available on the issue of counterfeit medicines in South Africa. This may, in part, be due to the fact that nobody knows the exact extent of the counterfeit medication problem, as it’s difficult to detect, investigate and quantify (Chowles, 2017). However, the problem is more prevalent in developing countries, where law enforcement and regulations are lax (Chowles, 2017). A South African article indicated that the country is increasingly being targeted by traffickers and is more vulnerable than its neighbors due to relatively high rates of online purchases (Knudsen & Nickels, 2015). The article indicated that awareness and mitigation efforts to curb the problem is much better in other African countries where levels of the problem are higher compared to South Africa (Knudsen & Nickels, 2015). However, considering that only 1 in 4 South Africans are aware of the existence of counterfeit medicines (Knudsen & Nickels, 2015), education programs would be an important first step in curbing the problem. Knudsen & Nickels (2015) indicate that South Africa is targeted by counterfeit medicine traffickers because of its long coastline, well-developed air transit infrastructure, high purchasing power, and frequent use of online pharmacies relative to neighboring countries (Knudsen & Nickels, 2015). Therefore, efforts to curb the problem will also need to consider these factors. The article states that government interventions, public awareness drives, and mobile technology campaigns in other African countries like Nigeria, Kenya and Ghana have been quite successful (Knudsen & Nickels, 2015) and may be worth considering in South Africa.

### Study limitations

The target population of this study was practicing veterinarians in the City of Tshwane Metropolitan Municipality and therefore the findings may not be generalizable to the whole of South Africa. This limitation notwithstanding, the results from this study offer valuable information regarding antimicrobial prescription practices and attitudes of veterinarians towards antimicrobial use and prescription.

## Conclusions

This study investigated prescription practices and attitudes towards AMR among veterinarians in the City of Tshwane Metropolitan Municipality, South Africa. Antimicrobials were emphasized in one or more courses both at pre-clinical and clinical levels with pharmacologists and clinical pharmacologists being responsible for most of the training. In addition, veterinarians consulted textbooks/drug handbooks, peer reviewed scientific literature, pharmaceutical companies and attended continuing professional education courses to get information on antimicrobial prescription and use. Decisions to prescribe antimicrobials depended largely on clinical presentation of the patient, while the choice of antimicrobials depended on cost, route of administration, and risk of potential adverse drug reactions. A number of veterinarians were of the view that antimicrobial prescriptions were done judiciously and that they did not over prescribe and neither did their colleagues. Although antimicrobial prescription policies were widely adopted, there is room for improvement. Therefore, we recommend a drive for veterinary practices to adopt antimicrobial prescription policies which in turn will promote judicious use of antimicrobials. However, addressing the inappropriate use of antibiotics will require a multifaceted approach guided by findings from surveillance programs and research.

## Supplemental Information

10.7717/peerj.10144/supp-1 Supplemental Information 1 Survey Questionnaire.

10.7717/peerj.10144/supp-2 Supplemental Information 2 Raw Dataset. All responses from the study survey questionnaire.

## Additional Information and Declarations

### Data Availability

The following information was supplied regarding data availability: The raw data are available in a Supplemental File.
