# Supplementary material for: Antibiotic prescription practices and attitudes towards the use of antimicrobials among veterinarians in the City of Tshwane, South Africa
Source: PeerJ. 2021 Jan 13;9:e10144. doi: 10.7717/peerj.10144 (PMC7811296; doi:10.7717/peerj.10144)
Supplement: Supplemental Information 1 [file peerj-09-10144-s001.docx]

Investigation of Opinions and Antibiotic Prescription Practices Among Veterinarians in Gauteng

**INTRODUCTION LETTER**

**Investigation of Opinions and Antibiotic Prescription Practices Among Veterinarians in Gauteng**

You are invited to participate in a survey of veterinarians that is part of research into the use of antimicrobials and prescription practices in veterinary medicine. We are requesting both small and large animal veterinarians to complete a questionnaire to collect information on antibiotic prescription practices and opinions on antimicrobial resistance. Your participation in this study is important and will help us better understand antimicrobial use in small and large animal veterinary practices. All responses are anonymous and completely confidential. The information that you provide in this questionnaire will not be made available to third parties. Participation in this study is entirely voluntary.

Thank you for your assistance

For any questions or concerns please contact:

Nenene Qekwana

Lecturer

University of Pretoria,

cnr Lynnwood Road and Roper Street,

Hartfield South Africa.

Email: [Nenene.Qekwana@up.ac.za](mailto:Nenene.Qekwana@up.ac.za)

Dr James W.Oguttu, Ph.D

College of Agriculture and Environmental Sciences

Department of Agric & Animal Health

UNISA Florida Campus

E-mail: [joguttu@unisa.ac.za](mailto:joguttu@unisa.ac.za)

**CONSENT FORM**

Nenene Qekwana

University of Pretoria

Email: Nenene.Qekwana@up.ac.za

You have been invited to take part in a research survey investigating the antimicrobial prescription practices among veterinarians and opinions regarding antimicrobial resistance. The survey will take approximately 15-20 minutes to complete. Your participation in this survey is important and will allow us to better understand the link between prescription practices and opinions regarding development of antimicrobial resistance. Taking part in this study is completely voluntary. If you choose to participate in this study, you can withdraw at any time. Your responses will be kept strictly confidential and anonymous. Any reports or publications that result from this research will be done at an aggregated level.

If you have questions or want a copy or summary of this study’s results, you can contact us at the email address above. If you have questions about your rights as a participant, you may contact the research ethics office at the University of Pretoria at 012 356 3084 or 012 356 3085. Please feel free to print a copy of this consent page to keep for your records.

- I have read the above information and I agree to participate in this study

**DEMOGRAPHICS**

1). What is your gender?

- Male
- Female

2). What city do you work in? ____________________________________________________

3). Is your veterinary practice:

- Small Animal
- Large Animal
- Equine
- Mixed

4). What type of veterinary facility do you practice at?

- Primary Care
- Referral
- Veterinary Hospital
- Charity Clinic
- Academic

5). How long have you worked at your practice? ______________________years

6). What is the total number of veterinarians employed at your facility or practice? ___________

7). How many hours per week do you work? ________________________________________

8). What year did you graduate with your veterinary degree? ____________________________

**VETERINARY EDUCATION**

9). What was the emphasis on antibiotics in your veterinary school education (non-clinical years)?

- a. Topic was not covered
- b. Light emphasis
- c. Covered thoroughly in one course
- d. Emphasized in multiple courses

10). What was the emphasis on antibiotics in your veterinary school education (clinical years)?

- a. Topic was not covered
- b. Light emphasis
- c. Covered thoroughly in one course
- d. Emphasized in multiple courses

11). What was the background of the person primarily responsible for your education on antibiotics during your veterinary education? (Please select all that apply)

- a. Clinical pharmacist
- b. Clinical microbiologist
- c. Clinician
- d. Pharmacologist/clinical pharmacologist
- e. Toxicologist
- f. Don’t know what his/her background was

12). Do you hold any additional post graduate qualifications?

- a. No
- b. Yes
- If yes, please list your post graduate qualifications below _________________________________________________________________________

**ANTIMICROBIAL PRESCRIPTION PRACTICES**

13). What are the main sources that you use to receive current information on antimicrobials and their use? (Please select all that apply)

- a. Practice policy
- b. Pharmaceutical companies
- c. Veterinary Medicine Directorates
- d. Peer reviewed scientific literature
- e. Textbook/Drug handbook
- f. Continuing Professional Development courses
- g. Other (Please specify) ____________________________________________________

14). Can you prescribe antibiotics without supervision, approval, or additional oversight?

- a. Yes
- b. No (Please explain) ______________________________________________________
- Never

15). Does your veterinary facility or practice have a policy concerning antibiotic prescription?

- a. Yes
- b. No

16). On Average, how often do you prescribe antibiotics?

- a. Multiple times per day
- b. Once per day
- c. Once every two days
- d. Once per week
- e. Once every two weeks
- f. Once per month
- g. Once every two to four months
- h. Quarterly
- i. Biannually
- j. Annually

17). Is there any antibiotic that you do not feel comfortable prescribing?

- a. No
- b. Yes, please explain below _________________________________________________________________________

**FACTORS POTENTIALLY ASSOCIATED WITH PRESCRIPTION PRACTICES**

18). Do any of the factors below affect your decision when choosing to prescribe an antibiotic to a patient? (Please select all that apply)

- a. Cost of antibiotic
- b. Client insurance
- c. Client expectations
- d. Route of administration
- e. Frequency of patient visits
- f. Risk of potential adverse drug reaction
- g. Other (Please specify below) _________________________________________________________________________

19). You always rely on clinical signs and symptoms when prescribing an antibiotic?

- a. Strongly agree
- b. Agree
- c. Neither agree nor disagree
- d. Disagree
- e. Strongly disagree

20). You rely on laboratory results before prescribing an antibiotic?

- a. Strongly agree
- b. Agree
- c. Neither agree nor disagree
- d. Disagree
- e. Strongly disagree

**OPINIONS ABOUT PRESCRIPTION PRACTICES**

21). What are your feelings concerning antibiotic prescription at your facility or practice?

- a. All antibiotics are under-prescribed
- b. Some antibiotics are under-prescribed (Please list them below)

____________________________________________________________________________

- c. All antibiotics are appropriately prescribed
- d. Some antibiotics are over-prescribed (Please list them below)

____________________________________________________________________________

- e. All antibiotics are over-prescribed

22). Do you feel like you sometimes over-prescribe antibiotics?

- a. No
- Yes

23). Your colleagues over-prescribe antibiotics?

- a. Strongly agree
- b. Agree
- c. Neither agree nor disagree
- d. Disagree
- e. Strongly Disagree

24). Veterinarians at your practice or facility always comply with antibiotic prescription policies.

- a. Strongly agree
- b. Agree
- c. Neither agree nor disagree
- d. Disagree
- e. Strongly disagree

25). Antibiotic prescription policies are contributing to a change in the frequency of antimicrobial resistance at your facility or practice?

- a. Strongly agree
- b. Agree
- c. Neither agree nor disagree
- d. Disagree
- e. Strongly disagree

**OPINIONS ABOUT ANTIMICROBIAL RESISTANCE**

26). Improper use of antibiotics contributes to selection for antimicrobial resistance.

- a. Strongly agree
- b. Agree
- c. Neither agree nor disagree
- d. Disagree
- e. Strongly disagree

27). How does improper use of antibiotics affect selection for antimicrobial resistance?

- a. It does not affect selection for antimicrobial resistance
- b. Improper use of antibiotics affects selection for antimicrobial resistance in the following ways: ____________________________________________________________________

28). Improper prescribing habits among your colleagues is affecting the selection for antibiotic resistance in your facility.

- a. Strongly agree
- b. Agree
- c. Neither agree nor disagree
- d. Disagree
- e. Strongly disagree

29). There has been an increase in the number of cases of antimicrobial resistance at your facility or practice.

- a. Strongly agree
- b. Agree
- c. Neither agree nor disagree
- d. Disagree
- e. Strongly disagree

30). In your opinion, what percentage of your clients are compliant with the instructions for prescribed antibiotics? __________

Thank you for completing this survey.
